# Supraclavicular Flap for Severe Post-burn Neck Contracture in Children

**DOI:** 10.7759/cureus.12910

**Published:** 2021-01-25

**Authors:** Terrence Jose Jerome, Vanathi Sabtharishi, Thirumagal SK

**Affiliations:** 1 Orthopaedics, Hand and Reconstructive Microsurgery, Olympia Hospital and Research Centre, Trichy, IND; 2 Department of Microbiology, K.A.P.Viswanatham (KAPV) Government Medical College, Trichy, IND; 3 Trauma, Olympia Hospital & Research Centre, Trichy, IND

**Keywords:** postburn contracture, neck, children, supraclavicular flap, aesthetic, functional outcome

## Abstract

The neck is essential and vital for all head movements and performing daily functional activities. The second-degree deep dermal and full-thickness burns causing anterior neck contracture restricts movement and if untreated develop deformities, in the oral cavity, eyes, posture, and chin growth and development, especially in children. Neck contracture results in kyphoscoliosis, lower lip seal resulting in impaired vision, balance, swallowing, feeding, and speech as well as social stigma, depression, and embarrassment. The treatment for post-burn anterior neck contractures is contracture release and reconstruction with skin grafts (split and full-thickness), axial pattern flaps, perforator propeller flaps, microvascular free flaps, tissue expansion, prefabrications, and skin substitutes. In addition to functional and esthetic recovery, post-surgery social and vocational rehabilitation is essential for children. We report a ten-year-old boy with severe anterior neck post-burn contracture managed with unilateral supraclavicular flap and residual areas with a split-thickness skin graft.

## Introduction

Post-burn contractures in the neck are disfiguring scars resulting in abnormal lip competence, facial expression, and decreased neck movements. It affects the growth and shape of the mandible in children. Post-burn neck contracture management aims to improve the neck extension angle, contracture release, and resurfacing the defects. The Onah classification describes the neck contractures as type 1-3 with a,b,c as subdivisions [1]. Type 1 is mild contracture. The patient can flex the neck as well as bring the neck and jaws to the anatomical position while erect. Also, limited extension away from the anatomical position (>90°) is possible with the inability to view an object located on the ceiling (180° to the erect patient) in the center of the visual field].

Type 2 is moderate contracture. Patients with this type of contracture could flex the neck and bring the neck and jaws to the anatomical position while erect. Attempts at extension away from the anatomical position resulted in a significant pull at the (uninvolved) lower lip. The extension (cervicomental) angles in this type were <90°.

Type 3 is severe anterior mentosternal contracture. The patient's neck is contracted in the flexed position, and the chin (less frequently the lower lip) is occasionally restrained down to the anterior trunk. The patient is unable to reach the anatomical position of the neck and jaws. In the attempt, the superior limbus of the unaffected eye was covered, and the inferior limbus of the unaffected eye was seen. The attempt also usually pulled on the (uninvolved) lower lip.

The classifications' subdivisions are 1) a-narrow band 2) b-broad band and 3) c-broad band with insufficient adjacent supple neck skin.

The surgical release and resurfacing may be single staged or multistage. The need for prefabrication and expanders is based on the width of the contracting segments and the availability of surrounding supple skin [2,3]. In addition to the neck contractures these patients also have shoulder stiffness, compensatory kyphosis, visual field deficits, lower lip ectropion, incomplete oral occlusion with saliva drooling, feeding and swallowing difficulties, unable to sleep, poor posture, depression, school dropouts, restricted social gathering and overall despondence [1-4].

The surgical planning needs to be multicentric focusing on correction of various deformities associated with post-burn contractures and social and vocational rehabilitation.

We report a ten-year-old boy with Onah type 3c post-burn contracture involving the entire anterior neck, severe mento sternal bands, and associated elbow and axillary contractures. We did a single-stage contracture release and covered the anterior neck with a unilateral supraclavicular flap and residual raw areas with split skin grafts. We corrected the contracture. The boy had a good esthetic and functional outcome at the follow up with returning to school and daily activities at the end of four months after the surgery.

## Case presentation

**Figure 1:**
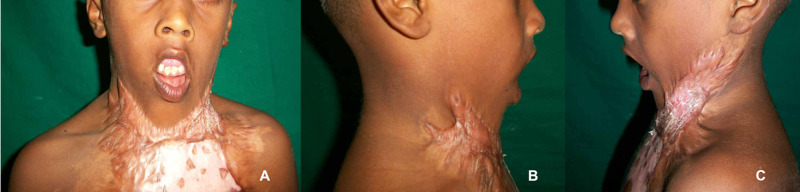
Clinical picture A- A ten-year-old boy with severe post-burn neck contracture and disfiguring scar involving the anterior neck and anterior chest wall involvement. He has also lip incompetence with lower lip ectropion; B- The cervicomental angle is very narrow and has no neck movements; C- Bowed down head

A ten-year-old boy sustained burns to his neck, left axilla, elbow, and chest wall at two years. He had initial treatment with split-thickness skin grafts and subsequent follow up with a soft cervical collar. The parents noticed gradual worsening of the deformity where the feeding and sleeping became difficult. He dropped out of school because of the disfiguring scar and facial abnormalities. We noted Onah type 3c severe anterior neck contracture, lower lip ectropion, drooling, left axilla and elbow contractures, inframammary contractures, hypertrophic scar tendency, and inadequate eye closure. (Figure 1)

**Figure 2:**
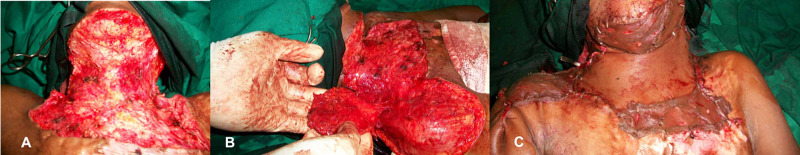
Intraoperative pictures A- The contracture is released under general anesthesia to achieve satisfactory neck extension; B-The supraclavicular artery is identified in the medial half of the flap by transillumination carefully preserved as the dissection continued medially towards the vessel's origin usually from the superficial transverse cervical artery; C- The anterior neck unit defect is covered with a supraclavicular artery flap. A split-thickness skin graft is applied to the submental defect, donor site, and the anterior chest wall.

We preferred the supraclavicular flap because of its reliability, anatomically similarity and proximity to the defect, good texture (thin flap), ease to harvest and rotate to resurface the large size neck defect with minimal or no donor site morbidity. But we faced a challenge in intubating our case because of the severe neck contracture. With the help of a fiberoptic laryngoscope, orotracheal intubation was done with specific difficulty. We performed the anterior scar contracture release in the supraplatsymal plane, excised the cervical fascia's investing layer using scalpel and electrocautery. We extended the release beyond the mid-axial plane at the hyoid bone level to release the submental unit and achieved full neck extension and lateral rotation. We measured the defect and elevated the supraclavicular flap (20x12 cm) with deltoid fascia sutured to the flap edges to prevent shearing. We identified the supraclavicular axial vessel and traced it up to its origin from the transverse cervical artery and rotated the flap through 180° and brought over the defect. We checked again for the full neck extension and lateral flexion before flap final suturing (Figure 2).

**Figure 3:**
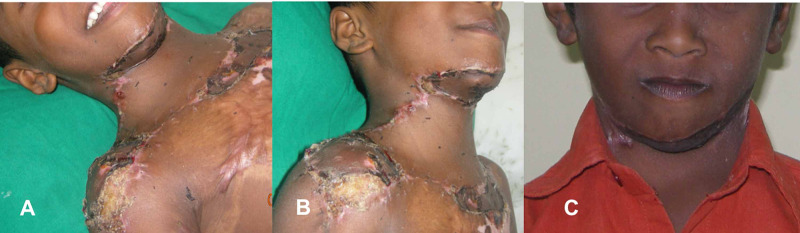
Follow up pictures The immediate and late follow-up pictures with good neck contour, color match, lip seal, and a normal range of neck movements. Also, a remarkable improvement in function and esthetics is achieved at 24 months of follow-up A- Side view ( 12 weeks follow up); B- Lateral view ( 12 weeks follow up); C- Frontal view ( 24 months follow up)

Once we found satisfactory flap reach and neck movements, we resurfaced the defect using 3/0 non-absorbable ethilon (Johnson & Johnson Ltd, India). We applied a split-thickness graft to the donor site, submental defects, chest wall with a tie over the dressing. This patient also had axillary contracture release and a split-thickness graft. We gave well-padded postoperative dressing for the neck and axilla and allowed feeding, sitting up, and walking in the subsequent days. We started tolerable neck flexion-extension and rotation movements in the first week after the surgery and shoulder mobilization during the second week. We removed the sutures two weeks after the surgery and applied a soft cervical collar to promote and maintain neck hyperextension of the neck for 12 weeks. We used silicon gels for the suture lines and gave pressure garments to the left axilla and elbow contractures. Postoperative neck support/splint was taken off and applied between the therapy. The flap settled well and the patient went to school four months after the surgery with comfort and full confidence (Figure 3).

The split-thickness graft over the donor site, chest wall, axilla had multiple raw areas settled with dressing and compression garments. The neck surgery spontaneously corrected the lower lip ectropion and eye closure, where he can close his lips and eyes voluntarily and freely. At the final follow of 24 months, he had useful neck extension, remarkable esthetic and functional outcome. He had patchy hypertrophic scars over the deltoid region, axilla and elbow and did not require any interventions. 

## Discussion

Severe post-burn neck contracture in children is always associated with severe unsightly disfigurations, ectropion of eyelids and lips, and considered as a social stigma depriving them of education and public gatherings [1-4]. Surgical reconstruction is mandated to rehabilitate back to their normal life and free from social embarrassments. The principle behind reconstructive surgeries includes improvement in neck extension angle, release and resurface neck contractures, and platysmaplasty to prevent recurrence and deepen the cervicomental angle [2-4]. Majority of cases may have intubation impracticable, and few can be intubated without much difficulty before contracture release [1]. Also, difficulty with intubation is anticipated when the distance between the chin and thyroid prominence is less than 6 cm in the adult [1]. Fiberoptic assisted intubation is the recommended mode for airway management in difficult intubation cases. 

Onah classification of scar contracture described the severity and guided the reconstructive options [1]. Type 1 requires Z plasty and skin grafts; type 2 needs excisional release and local transposition flaps; type 3 necessitates skin grafts in addition to excision and contracture release [5]. 

Lamberty described supraclavicular flap as an axial pattern flap in 1979 [6]. Since then, various authors have expressed their flap modifications and extended their uses in resurfacing face, neck, and anterior chest wall reconstructions [7,8]. The supraclavicular flap is a thin flap that can be pre-expanded, advanced as a tunneled flap or propeller flap. This flap provides wide coverage of the post-burn scar contractures defects in the neck and has inherited elasticity postoperatively to achieve good esthetic and functional outcomes [9,10]. The flap can be rotated at 130°-180° for inset into the submental and anterior neck units without cervical sagging, dog-ear, and other flap related complications [10]. 

The supraclavicular flap has an 86.7-90.24% of success rate in reconstructing the neck contractures [9-11]. A donor defect of less than 10 cm is managed with primary suturing, and more than 10 cm width requires skin grafting. An island flap provides 4-5 cm greater reach than the pedicle flap which is advantageous in severe neck contractures. Further coverage of the contracture released defects are managed by ligating transverse cervical artery and supercharging with posterior circumflex humeral vessels [12]. The division of the clavicular head of sternocleidomastoid increases the retrograde flap drainage and improves the flap sensibility by preserving the middle suprascapular nerve within the pedicle [13]. 

The supraclavicular flap is robust in its application. It can be prefabricated with thoracoacromial vessels, expanded as a cervicoacromial flap, and used as an island flap in the post-radiated neck reconstruction [13,14]. Moreover, the procedure is single staged with the donor site well hidden beneath garments. It may exhibit minimal debilitating scar hypertrophy and shoulder stiffness in the immediate follow-up. The postoperative neck hyperextension splints, physiotherapy, and neck movement training are well tolerated by this procedure. Less than 10% of the flap faces complications such as partial or total failure where splint skin grafts or free tissue transfers can be an alternative. 

Split thickness skin grafts (meshed and non-meshed) contracted 40.5-51.5 % to mean wound size in the postoperative period [15]. The recurrence is 62-81%, and they have a poor color match [16]. Supraclavicular flaps do better in restoring the cervicomental angle, chin projection, and good postoperative neck movements [17]. 

Free tissue transfers such as free scapular flaps, radial flap, groin flap, free parascapular flaps, and free thin anterolateral thigh flaps have reported a good color match, contour, and functional outcomes in post-burn neck contractures [18,19]. Longer operating time, microsurgical expertise, flap failures, suboptimal color match, and bulkiness limits free tissue transfers in post-burn scar contractures in the neck [20]. 

Our case is a ten-year-old boy who had Onah type 3c post-burn neck contracture with bowed down head, oral incompetence, inadequate eye closure, psychological stress, school dropout, embarrassments in a public appearance, difficulty in feeding, and drooling, sleep disturbances, and overall daily activities. We aimed for a single staged procedure considering the unsightly disfiguration, poor esthetic, functional, and psychological crippling caused by the contracture. Because of a limited range of flexion and extension, intubation was difficult in our case. Fiberoptic assisted intubation was successfully done. We raised a unilateral supraclavicular flap measuring 20x12 cm and covered the defect artery, the contracture release, and scar removal. The patient recovered well in the postoperative period. He regained good lip competence, eye closure, lower lip seal, and feeding with no drooling. At four months, he went back to school, started to play, and is leading a normal life for a child of his age. 

The supraclavicular flap is an ideal flap for severe post-burn neck contractures which can be harvested close to the neck to provide excellent color and texture characteristics. It covers the large neck defects by its virtue of stretch postoperatively. The thin and pliable skin allows a good range of neck movements with minimal or no donor site morbidity. It does not require pre-expansion or microsurgical expertise. 

## Conclusions

The supraclavicular flap is a reliable thin flap, with a great arc of rotation to cover large defects from severe neck contractures. The flap elevation is quick and straightforward performed as a single staged reconstructive procedure in children. This flap matches the neck skin contour, color and gives good esthetic and functional outcomes. The children with severe neck contractures have to be treated with a thorough plan such as they have to be rehabilitated soon to make them lead a normal life like others. Social stigma, depression, and poor feeding because of deformity can be managed by early surgical intervention and postoperative splints. Social rehabilitation should go in hand with the surgical plan for growing children. 
